# A T3SS Regulator Mutant of *Vibrio alginolyticus* Affects Antibiotic Susceptibilities and Provides Significant Protection to *Danio rerio* as a Live Attenuated Vaccine

**DOI:** 10.3389/fcimb.2020.00183

**Published:** 2020-04-28

**Authors:** Shihui Zhou, Xueting Tu, Huanying Pang, Rowena Hoare, Sean J. Monaghan, Jiajun Luo, Jichan Jian

**Affiliations:** ^1^Shenzhen Institute of Guangdong Ocean University, Shenzhen, China; ^2^Fisheries College, Guangdong Ocean University, Zhanjiang, China; ^3^Guangdong Provincial Key Laboratory of Pathogenic Biology and Epidemiology for Aquatic Economic Animals, Zhanjiang, China; ^4^Guangdong Key Laboratory of Control for Diseases of Aquatic Economic Animals, Zhanjiang, China; ^5^Key Laboratory of Experimental Marine Biology, Institute of Oceanology, Chinese Academy of Sciences, Qingdao, China; ^6^Laboratory for Marine Biology and Biotechnology, Qingdao National Laboratory for Marine Science and Technology, Qingdao, China; ^7^Institute of Aquaculture, University of Stirling, Stirling, United Kingdom

**Keywords:** *Vibrio alginolyticus*, live attenuated vaccine, type III secretory system, tyeA, regulator

## Abstract

*Vibrio alginolyticus* is a major cause of Vibriosis in farmed marine aquatic animals and has caused large economic losses to the Asian aquaculture industry in recent years. Therefore, it is necessary to control *V. alginolyticus* effectively. The virulence mechanism of *V. alginolyticus*, the Type III secretion system (T3SS), is closely related to its pathogenicity. In this study, the T3SS gene *tyeA* was cloned from *V. alginolyticus* wild-type strain HY9901 and the results showed that the deduced amino acid sequence of *V. alginolyticus tyeA* shared 75–83% homology with other *Vibrio* spp. The mutant strain HY9901Δ*tyeA* was constructed by Overlap-PCR and homologous recombination techniques. The HY9901Δ*tyeA* mutant exhibited an attenuated swarming phenotype and an ~40-fold reduction in virulence to zebrafish. However, the HY9901Δ*tyeA* mutant showed no difference in growth, biofilm formation and ECPase activity. Antibiotic susceptibility test was observed that wild and mutant strains were extremely susceptible to Amikacin, Minocycline, Gentamicin, Cefperazone; and resistant to oxacillin, clindamycin, ceftazidime. In contrast wild strains are sensitive to tetracycline, chloramphenicol, kanamycin, doxycycline, while mutant strains are resistant to them. qRT-PCR was employed to analyze the transcription levels of T3SS-related genes, the results showed that compared with HY9901 wild type, Δ*tyeA* had increased expression of vscL, vscK, vscO, vopS, vopN, vscN, and hop. Following vaccination with the mutant strain, zebrafish had significantly higher survival than controls following infection with the wild-type HY9901 (71.2% relative percent survival; RPS). Analysis of immune gene expression by qPCR showed that vaccination with HY9901Δ*tyeA* increased the expression of IgM, IL-1β, IL-6, and TNF-α in zebrafish. This study provides evidence of protective efficacy of a live attenuated vaccine targeting the T3SS of *V. alginolyticus* which may be facilitated by up-regulated pro-inflammatory and immunoglobulin-related genes.

## Introduction

*Vibrio alginolyticus*, a Gram-negative motile rod bacterium, is widely found in seawater and estuaries in various regions of the world (Thompson et al., 2004; Jones et al., 2013) *V. alginolyticus* is highly pathogenic in marine animals (Ben Kahla-Nakbi et al., 2009; Jun et al., 2015) and is associated with diseases of fish, shellfish, shrimp, and coral (Chang et al., 2008; Sadok et al., 2013). It is also a potential zoonotic pathogenic bacteria and incurs great economic losses to the aquaculture industry with raised pressures for the industry to develop controls in recent years. Zoonotic infections can result from human food poisoning causing diarrhea and other symptoms (Li et al., 2009; Sganga et al., 2009). Therefore, it is of great importance to understand the pathogenic mechanisms of *V. alginolyticus* in order to develop an effective vaccine.

Type III secretory system (T3SS) is a highly conserved secretory system in several Gram-negative bacteria such as *Yersinia* spp., *Salmonella* spp., *Vibrio cholerae, Pseudomonas* spp. (Anand et al., 2003; Masato et al., 2014; Khavong and Lorena, 2016; Dorothea et al., 2017). When *V. alginolyticus* infects the host, the T3SS can secrete virulence proteins outside the cell or on the surface of the host cell, or even directly inject virulent proteins into the host cell, resulting in cell death (Pallen et al., 2005). Although the T3SS mechanism is usually conserved in Gram-negative pathogens, the functions of its regulator vary widely. Comparative genome analysis showed that the T3SS of *V. alginolyticus* was similar to that of *Vibrio parahaemolyticus* (Martinez-Argudo and Blocker, 2010), but little is known about the regulator TyeA of *V. alginolyticus* T3SS.

Until now, there was no related research on *tyeA* gene in *Vibrio alginolyticus*, and its function was still being explored. It has been found that TyeA is a regulator of T3SS and involved in regulation of effector proteins expression (Sundberg and Forsberg, 2003). Numerous studies have shown that the YopN-TyeA heterodimer plays a particularly important role in regulating the secretion of effector proteins in *Yersinia* (Iriarte et al., 1998; Cheng and Schneewind, 2000; Schubot et al., 2004; Ferracci et al., 2005). In order to understand the function of TyeA in T3SS, we first constructed a mutant of *tyeA* gene, studied the biology and pathogenicity of the HY9901Δ*tyeA* strain and the transcription levels of T3SS-related genes were then analyzed by qRT-PCR. In addition, we found that HY9901Δ*tyeA* mutant can be used as an effective live vaccine against *Vibrio alginolyticus* in zebrafish and induces the expression of zebrafish pro-inflammatory and immunoglobulin-related genes.

## Materials and Methods

### Bacterial Strains, Plasmid, and Experimental Fish

The bacterial strains, plasmids, and zebrafish used in this study are listed in Table 1. *V. alginolyticus* HY9901 was isolated from diseased red snapper (*Lutjanus sanguineus*) (Cai et al., 2007) in Zhanjiang Port, Guangdong Province. *E. coli* DH5 α, *E. coli* MC1061 (λ pir) (Rubirés et al., 1997), S17-1 (λ Pir) (Simon et al., 2019), and suicide plasmid pRE112 (Edwards et al., 1998) were preserved in our laboratory. Healthy zebrafish (*Danio rerio*) were purchased from Zhanjiang Aquatic Market, 3–4 cm long and ~0.2 g in weight. The zebrafish were tested by bacteriological recovery tests and kept in seawater in a circulation system at 28°C for 2 weeks before the experiment.

**Table 1:** Bacterial strains, plasmids, and experimental fish used in this study.

**Strains, plasmids**	**Relevant characteristics**	**Source**
*V.alginolyticus* HY9901	Wild type, isolated from diseased *Lutjanus sanguineus* off the Southern China coast	(Cai et al., 2007)
*E. coli* DH5α	supE44 ΔlacU169 (ϕ80 lacZDM15) hsdR17 recA1 gyrA96 thi-1 relA1	TakaRa
MC1061 (λpir)	lacY1 galK2 ara-14 xyl-5 supE44 λpir	(Rubirés et al., 1997)
pRE112	pGP704 suicide plasmid, pir dependent, oriT, oriV, sacB, C mr	(Edwards et al., 1998)
S17-1 (λpir)	T prSmrrecA thi pro hsdR-M+RP4:2-Tc: Mu: K m T n7 λpir	(Simon et al., 2019)
S17-1-pRE-ΔtyeA	S17-1 containing plasmid of pRE-Δ*tyeA*, C mr	This study
pMD18-T	Cloning vector, Ampr	TakaRa
Zebrafish	experimental fish, purchased from Zhanjiang Aquatic Market	Zhanjiang Aquatic Market

### Reagents and Primers

TIANamp Bacteria DNA Kit (Beijing, Tiangen Biotech Co., Ltd.); Easy PureTMQuick GelExtraction Kit and Easy PureTM Plasmid MiniPrepKit (Beijing, TransGen Biotech Co., Ltd.); pMD18-T vector, ExTaqDNA polymerase, Prime START MHS DNA polymerase, KpnI, SacI, and T4DNA ligase were all purchased from TaKaRa (Japan). The primers were synthesized by Sangon Biotech Co., Ltd.

### Cloning and Sequencing of the *tyeA* Gene From *V. alginolyticus* HY9901

A pair of primers tyeAP1-F/tyeAP1-R were designed as shown in Table 2 according to the *V. alginolyticus* gene sequence (GenBank Number: GU074526.1). PCR was performed in a Thermocycler (Bio-Rad, CA, USA) under the following optimized amplification conditions: an initial denaturation at 95°C for 5 min, followed by 35 cycles of 94°C for 30 s, 55°C for 30 s and 72°C for 30 s. Five microlitre of each amplicon was examined on a 1% agarose gel stained with ethidium bromide. The PCR product was recovered from the agarose gel to ligate into the pMD18-T vector and transformed into *E. coli* DH5α (Table 1). The inserted fragment was sequenced by Sangon Biological Engineering Technology & Services Co., Ltd. (Shanghai, China). Similarity analyses of the determined nucleotide sequences and deduced amino acid sequences were performed by BLAST programs (http://blast.ncbi.nlm.nih.gov /Blast.cgi) and aligned using the program Clustal-X (version 1.81). Protein analysis was conducted with ExPASy tools (http://expasy.org/tools/). Location of the domain was predicted using the InterProScan program (http://www.ebi.ac.uk/Tools/pfa/iprscan/).

**Table 2:** Sequences of primers used in this study.

**Primer name**	**Primer sequence(5^**′**^-3^**′**^)**	**Accession number**
tyeA-for	GGAATCTAGACCTTGAGTCGATATCTCGACCATCGCGCAA	MN328351
tyeA-int-rev	ATCTTCCCACGCTTCCTCTTCAACTTGATAAGCCATAATTCGTCC	
tyeA-int-for	GGACGAATTATGGCTTATCAAGTTGAAGAGGAAGCGTGGGAAGAT	
tyeA-rev	ACAGCTAGCGACGATATGTCAGGCCGGAGGTCATAGAGCT	
tyeA-up	CACATGAACTCGTTTCGGACTATT	
tyeA-down	TTTCTGGACGCAACAACTCTGA	
tyeAP1-F	ATGGCTTATCAAGTTTCTA	
tyeAP1-R	TCAATCCAACTCATCTTCC	
IL-6-F	GGTCAGACTGAATCGGAGCG	NM_001079833.1
IL-6-R	CAGCCATGTGGCGAACG	
IL-6R-F	GCATGTGCTTAAAGTATCCTGGTC	NM_001114318.1
IL-6R-R	TGCAAATTGTGGTCGGTATCTC	
IL-1β-F	TGGACTTCGCAGCACAAAATG	AY340959.1
IL-1β-R	GTTCACTTCACGCTCTTGGATG	
IL-8-F	GTCGCTGCATTGAAACAGAA	XM_001342570.2
IL-8-R	CTTAACCCATGGAGCAGAGG	
IgM-F	GTTCCTGACCAGTGCAGAGA	AF246193
IgM-R	CCTGATCACCTCCAGCATAA	
TNF-α-F	TAGAACAACCCAGCAAAC	NC_007130.7
TNF-α-R	ACCAGCGGTAAAGGCAAC	
rag-1-F	GAAGTATACCAGAAGCCTAAT	NC_007136.7
rag-1-R	TTCCATTCATCCTCATCACA	
TLR5-F	GAAACATTCACCTGGCACA	NC_007131.7
TLR5-R	CTACAACCAGCACCACCAGAATG	
c/ebpβ-F	GCCGTACCAGACTGCTCCGA	NC_007119.7
c/ebpβ-R	AGCCGCTTCTTGCCTTTCCC	
β-actin-F	ATGGATGAGGAAATCGCTGCC	NM_131031.1
β-actin-R	CTCCCTGATGTCTGGGTCGTC	
vscL-F	TACCACGGTGAGTGTAGTTC	ACY41051.1
vscL-R	CGTAACCGACTTCAGGGA	
hop-F	CTTCGCTTTCGGTTTGCT	KX245315
hop-R	AATACCATCCCACCCTGT	
vscO-F	GAGCTGGAAACATTAAGACA	ACY41065.1
vscO-R	TTGCTGCAACTGAACGAA	
vscK-F	GGCGTTATCTCCCGTTCC	ACY41050.1
vscK-R	CTCCGCCCACCATCAATA	
vopN-F	TGAACTCGTTTCGGACTA	ACY41067.1
vopN-R	ACTTTCTGGACTCGCACT	
vscN-F	TAGGCGAAGAAGGAATGG	ACY41066.1
vscN-R	GCGATAGAAGTGGCAACAA	
vopS-F	AGTTTTGGAAGTGTTAGCG	ACY41053.1
vopS-R	ACATTGCCTCTGTCATCG	
16S-F	TTGCGAGAGTGAGCGAATCC	NR_044825.2
16S-R	ATGGTGTGACGGGCGGTGTG	

### Construction of In-frame Deletion Mutant of *tyeA* Gene

According to the method previously described Zujie et al. (2019), Overlap extension PCR was applied to generate an in-frame deletion of the *tyeA* gene on the *V. alginolyticus* wild-type HY9901 chromosome. The in-frame deletion of *tyeA* in the *V. alginolyticus* was generated according to the method of Rubirés et al. (1997). For the construction of Δ*tyeA*, two PCR fragments were generated from HY9901 genomic DNA. The first fragment was amplified using primers tyeA-for (contains a KpnI site at the 5′-end) and tyeA-int-rev, whereas primers tyeA-int-for and tyeA-rev (contains a SmaI site at the 5′-end) were used to amplify the second fragment. Both fragments contained a 20 bp overlapping sequence and were used as templates for the subsequent PCR procedure, which used primers tyeA-for and tyeA-rev. The PCR product was ligated into suicide vector pRE112 (Cmr) to generate pRE-Δ*tyeA*. This recombinant suicide plasmid was transformed into *E. coli* MC1061λpir and subsequently S17-1λpir. The single crossover mutants were obtained by conjugal transfer of the resulting plasmid into *V. alginolyticus* HY9901. Deletion mutants were screened on 10% sucrose TSA plates. Its presence was subsequently confirmed by PCR and sequencing using primers tyeA-up and tyeA-down.

### Characterization of the Δ*tyeA*

#### Genetic Stability of Mutants HY9901ΔtyeA

HY9901Δ*tyeA* was inoculated onto a TSA plate and passaged blindly for 30 generations. In brief, a single colony was picked from Tryptic Soya agar (TSA) plates and cultured in Tryptic soya broth (TSB, HKM, Guangzhou, China) medium, shaking for 12 h, bacterial broth culture was streaked out on a TSA plate, and this process repeated 30 times. Its genetic stability was determined by the PCR method.

#### Growth Curve of Bacteria

The wild-type HY9901 strain and the Δ*tyeA* were cultured in TSB for 24 h. The HY9901 and HY9901 Δ*tyeA* were inoculated into TSB at the ratio of 1: 100 (OD_600_ = 0.5) at 28°C, with determination of OD_600_ every 2 h. This procedure was repeated three times per group.

#### Detection of Extracellular Protease Activity

According to the previously published method (Windle and Kelleher, 1997) wild-type strain HY9901 and HY9901Δ*tyeA* were coated on TSA plates coated with sterile cellophane, and cultured at 28°C for 24 h, washed with sterile PBS, centrifuged at 4°C for 30 min, and the supernatant filtered to obtain extracellular products. Inactivated sample (supernatant was boiled for 10 min) was used as a blank control.

#### Swarming Motility

Single colonies of wild-type strain HY9901 and HY9901Δ*tyeA* were selected by sterile toothpick and plated on TSA plates with agar content of 0.5%. Each group was incubated for 24 h at 28°C. The diameter of swarming circle was measured by Vernier calipers.

#### Detection of Biofilm Formation Ability Using Crystal Violet Ammonium Oxalate

With reference to the method described previously (Katharine and Watnick, 2003), wild strains HY9901 and HY9901 Δ*tyeA* (OD_600_ = 0.5) were transferred to a 96-well plate. Each well was inoculated with 200 μL of bacterium with 6 replicates per sample, the negative control was an inoculum of TSB only, and the culture temperature was 28°C. Samples were taken from the 96 well plate at 12, 24, 48, and 72 h, methanol fixed for 20 min, stained with Crystal violet ammonium oxalate dye for 15 min, rinsed with water and dried. Finally, 95% alcohol was added and incubated at room temperature for 30 min. OD_570_ was determined by Multimode Plate Reader(PerkinElmer EnSpire, EnSpire, Singapore) (OD_600_ = 1, bacterial concentration = 1 × 10^9^ cfu/mL).

#### Detection of Biofilm Formation Ability Using

##### Laser Scanning Confocal Microscope (LSCM)

The wild strain HY9901 and the mutant strain HY9901Δ*tyeA* (OD_600_ = 0.5) were diluted 50-fold, added to a glass bottom culture dishes (spec: type 28.2 mm, class diameter 20 mm) (Wuxi NEST, Wuxi, China) and statically cultured in a 28°C biochemical incubator for 24 h, gently washed three times with physiological saline, and then combined with 10% SYTO9 green. Bacteria were incubated with fluorescent dyes in the dark for 20 min, washed three times with saline, mounted in 40% saline-glycerol and observed by confocal microscopy (Zeiss, LSM710, Germany). The excitation wavelength was 488 nm, scanned from the bottom to the top of the biofilm, Z-section was 1 μm apart, and biofilm parameters—biomass and maximum thickness were determined. Three samples were made for each strain and the average amount calculated.

#### Dose Response Challenge Test (LD_50_)

The injection concentrations used for the dose response of wild-type strain HY9901 and Δ*tyeA* were 10^4^, 10^5^, 10^6^, 10^7^, and 10^8^ CFU/mL. A total of 330 fish were randomly divided into three groups (Table 3). The water temperature was adjusted to 28°C. The experiment was repeated three times. Five microlitre of bacterial solution was injected into fish by intramuscular injection. The control group was injected with equivalent volumes of PBS. Fish were monitored for 14 days or until no morbidities occurred. Specific mortality was determined by streaking head-kidney onto TSA. The LD_50_ of the mutant and wild strains was calculated by the Koch method (Reed and Muench, 1938).

**Table 3:** Experiment of LD_50_.

**Concentration (CFU/mL)**	**HY9901**	**Death rate (%)**	***ΔtyeA***	**Death rate (%)**	**Control (PBS)**	**Death rate (%)**
10^8^	10 × 3	90	10 x 3	66.7	–	–
10^7^	10 × 3	80	10 x 3	40	–	–
10^6^	10 × 3	60	10 x 3	10	–	–
10^5^	10 × 3	20	10 x 3	0	–	–
10^4^	10 × 3	20	10 x 3	0	–	–
0(PBS)	–	–	–	–	10 × 3	0

*Each experiment involved 110 zebrafish with 10 fish per group in three replicate tanks and the experiment was repeated three times (total fish = 330)*.

#### Antibiotic Susceptibility

The susceptibility patterns of the HY9901 and HY9901Δ*tyeA* strains to 30 different antibiotics were determined according to the disc diffusion method using TSA (Bauer et al., 1966), and the diameters of the inhibition zones were measured using Vernier calipers. Resistant, intermediate and susceptible phenotype determinations were based on previously published susceptibility paper guidelines (HANGWEI, S1100, China). The strains were inoculated onto TSA plates and allowed to absorb onto agar for 10 min, and antibiotic discs were added after 24 h of incubation at 28°C (Cai et al., 2016).

### Expression Analysis of T3SS-Related Genes

T3SS secretion was induced by Dulbecco's Modified Eagle Medium (DMEM) media, and strains HY9901 and HY9901 Δ*tyeA* were cultured for 12 h. The primers for T3SS related genes are shown in Table 2 (All primers were designed within the current study). The genes in this study were hop (Pang et al., 2018), vopN, vscN (Yonghong et al., 2016), vscO (Zhou et al., 2013), vopS, vscL, and vscK (Nguyen et al., 2000). 16S rRNA is used as an internal reference. According to the experimental method of Li et al. (2016), RNA was extracted, synthetic cDNA and real-time PCR were used to analyze the expression of T3SS related genes.

### Preparation of Vaccine and Vaccination

The Δ*tyeA* immune concentration of 10^5^ CFU/mL was selected by LD_50_ experiments, which has no mortality to zebrafish (Table 3). The mutant Δ*tyeA* was cultured in a shaking flask at 28°C for 18 h, washed and suspended in sterile artificial seawater, and the concentration of the bacterial solution was adjusted to 10^5^CFU mL^−1^ by spectrophotometer. The experimental fish were randomly divided into two groups with 80 fish in each group. The water temperature was adjusted to 28°C. Each fish was injected with 5 μL Δ*tyeA* bacterial solution (10^5^ CFU mL^−1^) by intramuscular injection and control fish were injected with 5 μL PBS per fish.

### Determination of Vaccine Efficacy

Fish were challenged 28 days post vaccination. Thirty fish were randomly selected from each group (in triplicate) and given intramuscular injection of 5 μL *V. alginolyticus* the wild-type HY9901 (1 × 10^8^ CFU mL^−1^). The water temperature was adjusted to 28°C and the number of moribund/mortalities was recorded for 14 days. Calculation of the relative percentage survival was performed at the end of the experiment (RPS (%) = (1-immunized group mortality / control group mortality) × 100%).

### Immune Gene Expression of Zebrafish Induced by HY9901Δ*tyeA* Vaccine

Liver and spleen samples were taken from three fish from each group, respectively, 1 day before challenge. Immune-related genes expression levels were detected with real-time qPCR. Primers for IL6, IL6R, IL-1β, IL-8, IgM, TNF-α, rag-1, TLR5, and c/ebpβ are shown in Table 2, and β-actin was used as internal reference. The procedures of RNA extraction, cDNA synthesis, real-time qPCR for analysis of immune gene expression were described by Li et al. (2016).

### Ethics Statement

All animal experiments were conducted strictly based on the recommendations in the “Guide for the Care and Use of Laboratory Animals” set by the National Institutes of Health. The animal protocols were approved by the Animal Ethics Committee of Guangdong Ocean University (Zhanjiang, China).

### Biosecurity

The bacteria protocols were approved by the Biosecurity Committee of Guang dong Ocean University (Zhanjiang, China).

### Statistical Analysis

The experimental data were analyzed by single factor analysis of variance (ANOVA) with SPSS17.0 software. ^**^indicates extremely significant difference compared with the control group (*p* < 0. 01). ^*^indicates significant difference compared with the control group (*p* < 0.05).

## Results

### Cloning of *tyeA* Gene and Construction of Mutant

**Figure 1:**
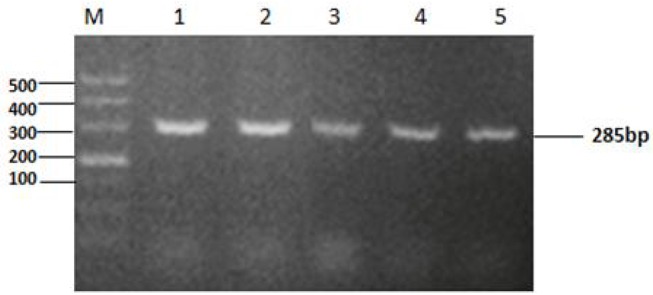
Cloning of *tyeA* gene. M: DL2000 marker. Lane 1-5:The 285bp fragment was amplified from genomic DNA of the wild-type strain HY9901 using primer pairs of tyeAP1-for/tyeAP1-rev (Lane 1, 2, 3, 4, 5:Tm = 56°C, 57°C, 58°C, 59°C, 60°C).

**Figure 2:**
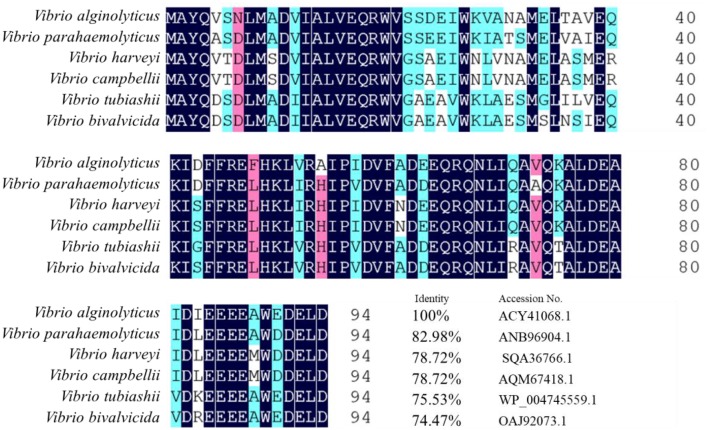
Homology comparison of *Vibrio parahaemolyticus* HY9901 T3SS Regulator Protein TyeA. *Vibrio alginolyticus* T3SS regulatory protein TyeA Accession No.ACY41068.1; *Vibrio parahaemolyticus* T3SS regulatory protein TyeA Accession No.ANB96904.1; *Vibrio harveyi* T3SS regulatory protein TyeA Accession No.SQA36766.1; *Vibrio campbellii* T3SS regulatory protein TyeA Accession NO.AQM67418.1; *Vibrio tubiashii* T3SS regulatory protein TyeA Accession No.WP_004745559.1; *Vibrio bivalvicida* T3SS regulatory protein TyeA Accession No.OAJ92073.1.

**Figure 3:**
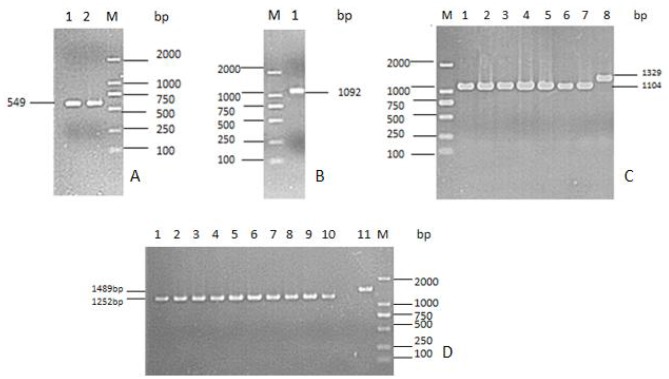
Construction and confirmation of the knockout mutant strain HY9901Δ*tyeA*. **(A)** M: DL2000 marker; Lane 1. The 549 bp upstream fragment amplified from genomic DNAs of the strain HY9901 using primer pairs of tyeA-for/tyeA-int-rev; Lane 2. The 543 bp downstream fragment amplified from genomic DNAs of the strain HY9901 using primer pairs of tyeA-int-for / tyeA-rev. **(B)** M: DL2000 marker; Lane 1. The 1,092 bp fragment amplified from genomic DNAs of HY9901Δ*tyeA* using primer pairs of tyeA-for/tyeA-rev. **(C)** M: DL2000 marker; Lane 1–7. The 1,104 bp fragment amplified from genomic DNAs of the HY9901Δ*tyeA* using primer pairs of tyeA-for/tyeA-rev; Lane 8. The 1,329 bp fragment amplified from genomic DNAs of the wild-type strain HY9901 using primer pairs of tyeA-for/tyeA-rev. **(D)** M: DL2000 marker; Lane 1–10. The 1,252 bp fragment amplified from genomic DNAs of HY9901Δ*tyeA*; Lane11. The 1,489 bp fragment amplified from genomic DNAs of the strain HY9901 using primer pairs of tyeA-up / tyeA-down.

The *tyeA* gene consists of an open reading frame of 285 bp (Figure 1), encoding 95 amino acids (aa) with a predicted molecular weight of 10.98 kDa (tyeA accession no. MN328351). The deduced amino acid sequence analysis of TyeA show it has high homology of 75–83% with other *Vibrio* spp. (Figure 2), with the highest homology shared with *V. parahaemolyticus* (83%). An untagged in-frame deletion mutant HY9901Δ*tyeA* was constructed by over-lap PCR and forward and reverse screening methods. The mutant was confirmed by inabilityto grow on TSA supplemented with chloramphenicol, and verified by PCR by generating a fragment of ~1,092 bp. Using PCR the genome of HY9901Δ*tyeA* and wild-type strain HY9901 by primer *tyeA*-up/*tyeA*-down, a 1,252 bp fragment was obtained for HY9901Δ*tyeA* and fragment of 1,489 bp was obtained for HY9901 (Figure 3).

### Characterization of the *tyeA*

#### Genetic Stability of Mutants

**Figure 4:**
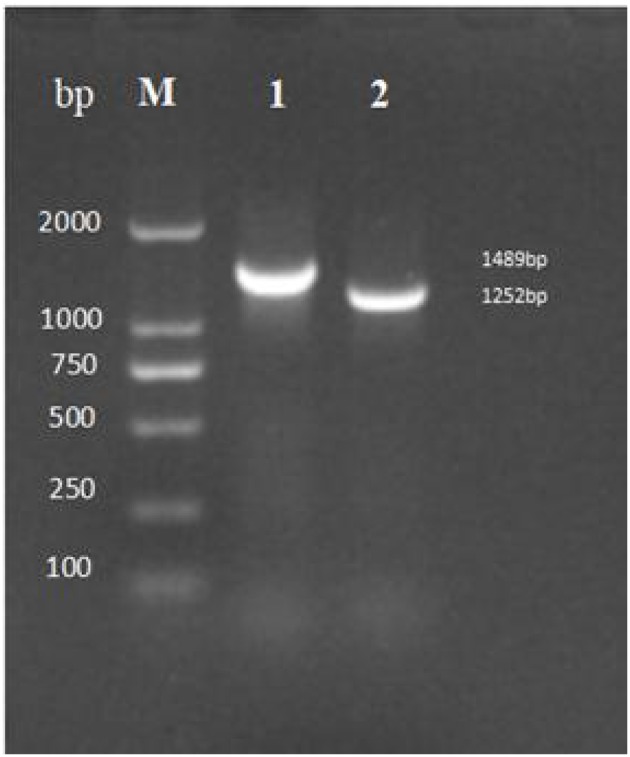
Genetic stability detection of HY9901Δ*tyeA* deletion mutant. M: DL2000 marker, Lane 1. A fragment of 1,489 bp is obtained for HY9901 using primer pairs of tyeA-up/tyeA-down. Lane 2. A fragment of 1,252 bp is obtained for HY9901Δ*tyeA* using primer pairs of tyeA-up/tyeA-down.

After 30 generations of continuous passages of mutant Δ*tyeA*, the PCR genome of HY9901Δ*tyeA* and wild-type strain HY9901 using primers *tyeA*-up/*tyeA*-down, a fragment of 1,489 bp was obtained for HY9901, and fragment of 1,252 bp was obtained for HY9901Δ*tyeA*. This shows that the wild-type strain HY9901 has 237 bp more than the mutant Δ*tyeA*, and the mutant Δ*tyeA* has deleted the gene tyeA, which can stabilize the inheritance (Figure 4).

#### Comparison of Growth Rates of Wild Type and Mutant Bacteria

**Figure 5:**
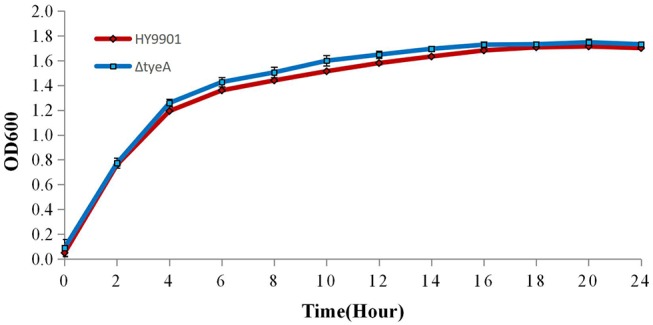
Growth rates of HY9901Δ*tyeA* and HY9901. Aliquots of cell culture were taken at various time points and measured for cell density at OD_600_.

The growth rate of wild-type strain HY9901 and HY9901Δ*tyeA* was similar and not statistically significant (*p* > 0.05) (Figure 5), the deletion of *tyeA* gene has no effect on the growth of *V. alginolyticus*. The exponential growth phase of the two bacterial strains was from 0 to 4 h, with growth stationary at 18 h, OD600 ≈ 1.7.

#### Extracellular Protease Activity

Extracellular proteases, as metabolites of bacteria, play an important role as virulence factors in the process of infecting the host. Extracellular proteins have a variety of protease activities, including lecithin, amylase, lipase, and casein. The extracellular protease activity was not significantly different between HY9901Δ*tyeA* and the wild-type strain HY9901 (*p* > 0.05) (Table 4).

**Table 4:** Comparison of biological characteristics between HY9901 and HY9901Δ*tyeA*.

**Characteristics**	**HY9901**	**HY9901Δ*tyeA***
Activity of ECPase(A_422_)^a^	1.03 ± 0.2	0.97 ± 0.2
Biofilm thickness(μm)^b^	60 ± 10	90 ± 20
Swarming (mm)^c^	34.9 ± 0.2	20.6 ± 0.4^**^
LD_50_(CFU mL^−1^)^d^	5.8 × 10^5^	2.6 × 10^7^^**^

*Values are mean ± standard deviation for three trials. Significant differences between HY9901 and HY9901ΔtyeA indicated by asterisk*.

** *p < 0.01*.

a *Bacteria were incubated in TSB for 18 h at 28°C*.

b *Bacteria were incubated in a glass bottom culture dish (NEST) for 24 h at 28°C*.

c *Swarming diameters were measured after 24 h incubation on TSA containing 0.3% agar plates*.

d *LD_50_were evaluated in healthy zebrafish with an average weight of 0.2 g*.

#### Detection of Biofilm Formation Ability

**Figure 6:**
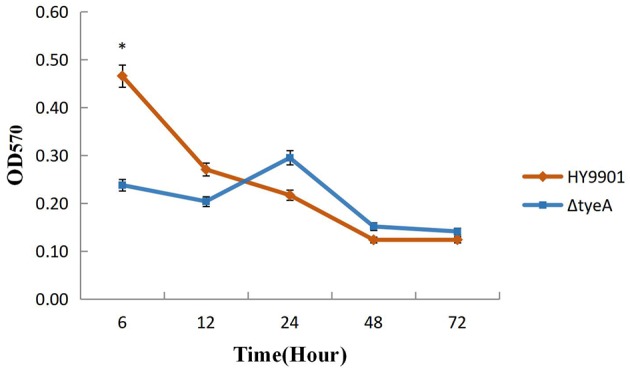
Measurement of biofilm by crystal violet ammonium oxalate. *indicates significant difference compared with the control group (*p* < 0.05). **indicates extremely significant difference compared with the control group (*p* < 0.01).

**Figure 7:**
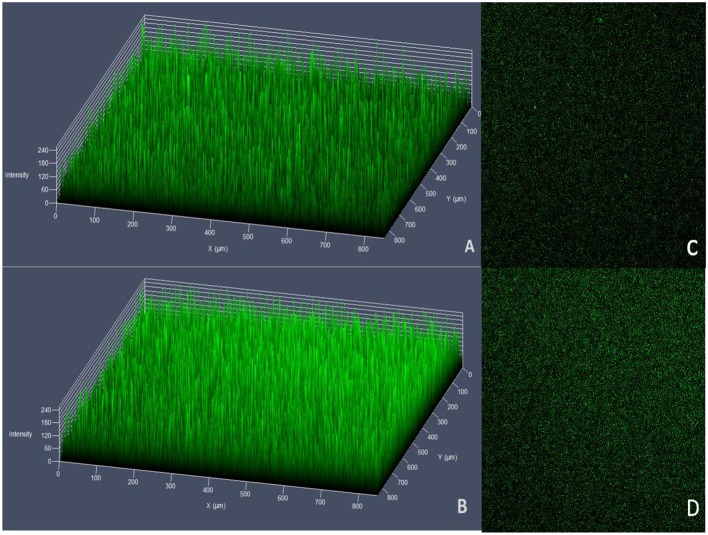
Measurement of biofilm by LSCM. **(A)** HY9901 2.5d diagram. **(B)** HY9901Δ*tyeA* 2.5d diagram. **(C)** HY9901 2d diagram. **(D)** HY9901Δ*tyeA* 2d diagram; HY9901 Biofilm thickness:60 ± 10 μm, HY9901Δ*tyeA* Biofilm thickness:90 ± 20 μm.

No significant differences were observed in biofilm formation at 24 h (*p* > 0.05) between wild strains and mutants of *V. alginolyticus* using the crystal violet ammonium oxalate assay (Figure 6) or by confocal microscopy (Figure 7 and Table 4). The biofilm fluctuates up and down within 72 h in crystal violet ammonium oxalate. The biofilm was thicker in HY9901 than in the mutant at 6 h (*p* < 0.05). After 12 h, the biofilm thickness was basically equal, and there was no significant change according to biological statistical analysis.

#### Swarming Motility

The wild-type strain HY9901 and HY9901Δ*tyeA* were inoculated on the swarming plate, and the results were as follows: the swarming circle of wild strain was 34.9 ± 0.2 mm, mutant was 20.6 ± 0.4 mm (Table 4). The swarming circle diameter of HY9901Δ*tyeA* was significantly smaller than that of wild strain (*p* < 0.01), indicating that the swarming ability of HY9901Δ*tyeA* was significantly weakened.

#### LD_50_ Determination

Zebrafish from quarantined stocks recognized as disease free (Xu et al., 2010) were used as models (Sullivan and Kim, 2008) to assess the virulence of the strain HY9901 and HY9901Δ*tyeA*. The results showed that the 50% lethal dose of HY9901Δ*tyeA* was 40 times higher than that of wild strain (Tables 3, 4). The symptoms of body surface hyperemia, abdominal redness and swelling and slow swimming were observed in the diseased fish. The results showed that the virulence of HY9901 Δ*tyeA* was significantly decreased when compared with the wild strain (*p* < 0.01).

### Antibiotic Susceptibility

Both wild and mutant strains were extremely susceptible to amikacin, minocycline, gentamicin, Cefperazone; resistant to oxacillin, clindamycin, ceftazidime, penicillin, ampicillin, caebenicillin, cefazolin, ceftriaxone, cephradine, piperacillin. Wild strains were sensitive to tetracycline, chloramphenicol, kanamycin, doxycycline, while mutant strains were resistant to them (Table 5).

**Table 5:** Drug sensitivity test results of the HY9901 and HY9901Δ*tyeA*.

**Antibiotic**	**Dose (μg)**	**Bacteriostatic circle diameter (mm)**			
		**HY9901**	**Sensitivity**	**Δ*tyeA***	**Sensitivity**
Cefperazone	75	0	R	0	R
Oxacillin	1	0	R	0	R
Clindamycin	2	0	R	0	R
Ceftazidime	30	0	R	0	R
Penicillin	10U	0	R	0	R
Ampicillin	100	0	R	0	R
Caebenicillin	100	0	R	0	R
Cefazolin	30	8.0 ± 0.2	R	0	R
Ceftriaxone	30	9.3 ± 0.3	R	0	R
Cephradine	30	0	R	0	R
Piperacillin	100	0	R	0	R
Cefuroxime	30	0	R	0	R
SMZ/TMP	23.75/1.25	0	R	0	R
Aboren	30	0	R	0	R
Vancomycin	30	0	R	0	R
Cephalexin	30	0	R	0	R
polymxinB	200IU	0	R	0	R
Norfloxacin	10	0	R	0	R
Ofloxacin	5	0	R	0	R
Ciprofloxacin	5	0	R	0	R
Amikacin	30	13.3 ± 0.3	I	10.0 ± 0.1	I
Minocyline	30	18.5 ± 0.2	S	14.3 ± 0.3	I
Tetracyline	30	13.5 ± 0.2	I	12.5 ± 0.2	R
Gentamicin	10	14.5 ± 0.1	I	12.5 ± 0.2	I
Furazolidone	300	10.5 ± 0.4	R	8.0 ± 0.1	R
Chloramphenicol	30	17.2 ± 0.3	S	0	R
kanamycin	30	14.1 ± 0.2	I	12.1 ± 0.2	R
Erythromycin	15	10.1 ± 0.2	R	0	R
Doxycycline	30	16.5 ± 0.3	S	10.6 ± 0.1	R
Cefperazone	30	14.2 ± 0.3	I	13.3 ± 0.3	I

*S(susceptible) I(intermediate) R(resistance)*.

### T3SS-related Gene Expression Analysis

**Figure 8:**
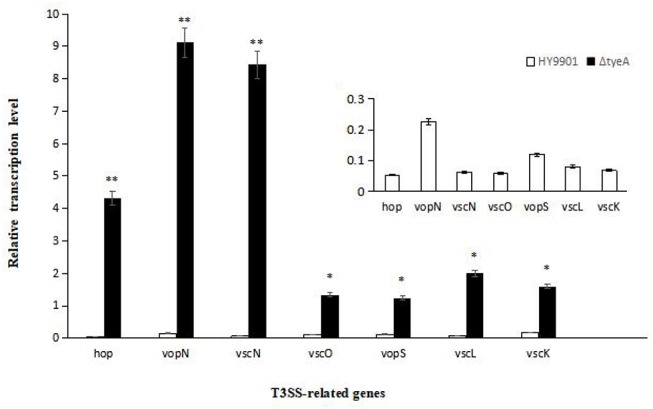
Expression of HY9901 and HY9901Δ*tyeA* T3SS-related genes induced by DMEM. Δ*tyeA* had significantly increased expression of *vscL, vscK, vscO, vopS* (*p* < 0.05), *vopN, vscN*, and *hop* (*p* < 0.01). * indicates significant difference compared with the control group (*p* < 0.05). ** indicates extremely significant difference compared with the control group (*p* < 0.01).

QRT-PCR was employed to analyze the transcription levels of T3SS-related genes including *vscL, vscK, vopN, vscO, vscN, vopS*, and *hop*. The results showed that compared with HY9901 wild type, Δ*tyeA* had significantly increased expression of *vscL, vscK, vscO, vopS* (*p* < 0.05), *vopN, vscN* and *hop* (*p* < 0.01) (Figure 8).

### Vaccine Efficacy

**Figure 9:**
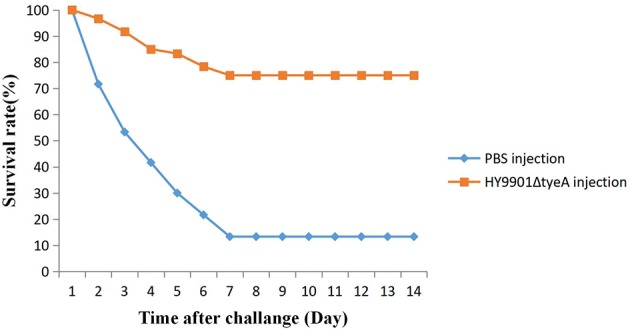
Survival in groups vaccinated with HY9901Δ*tyeA* and PBS following challenge with *Vibrio alginolyticus* HY9901.

Fish vaccinated with the mutant strain HY9901Δ*tyeA* were challenged 28 days post-vaccination with the wild strain. Within 14 days, the mortality rate in the control group injected with sterile PBS was 87%, the mortality rate in the injection immunization group was 25%, and the relative percentage survival was 71.2% (Figure 9).

### Immune Gene Expression of Zebrafish Induced by HY9901Δ*tyeA* Vaccine

**Figure 10:**
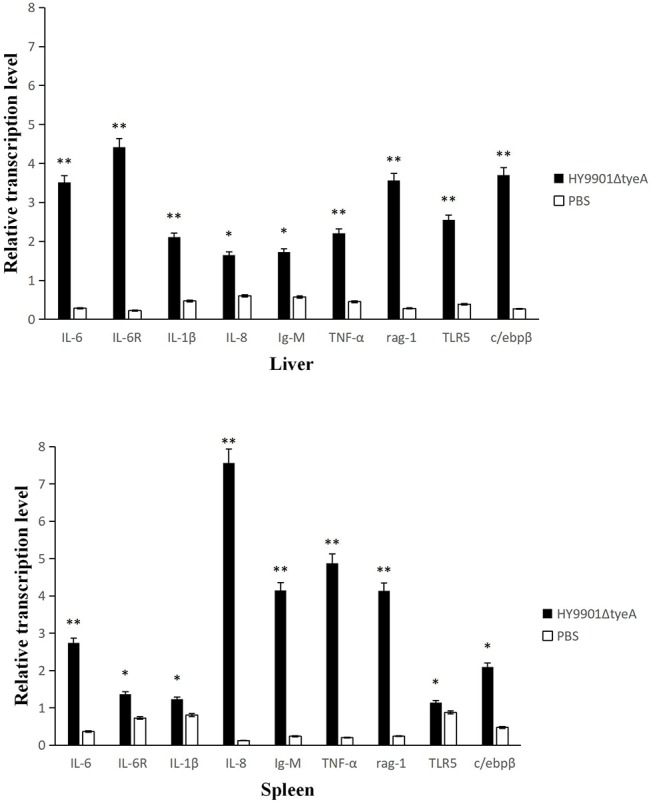
Comparative analysis of the expression of immune-related genes in liver and spleen of zebrafish given the live attenuated vaccine and unvaccinated zebrafish. The head kidney and spleen of grouper were sampled at 1 day before challenge, and the mRNA level of each immune-related gene was normalized to that of β-actin. Bars represent the mean relative expression of three biological replicates and error bars represent standard deviation.

Detection of immune gene expression in zebrafish immunized with HY9901Δ*tyeA* live attenuated vaccine was assessed by qPCR to analyze the transcriptional levels of pro-inflammatory and immunoglobulin-related immune genes. The results showed that, the group vaccinated with the mutant strain HY9901Δ*tyeA* had significantly increased expression of IL6, IL6R, IL-1β, TNF-α, rag-1, TLR5, c/ebpβ genes in liver and IL6, IL8, IgM, TNF-α, rag-1 genes in spleen compared to control fish injected with PBS (*p* < 0.01) (Figure 10).

## Discussion

At present, the interaction mechanisms between some fish pathogens and hosts have been studied in depth. Based on these studies, the development of efficient new vaccines has become highly desirable in aquaculture (Pang et al., 2016). Attenuated live vaccines are one of the more efficient vaccines, especially compared to inactivated vaccines due to their greater activation of cellular immunity. Currently, the construction of attenuated strains by gene knockout is an important method to obtain candidate attenuated live vaccines.

In the current study, we knocked out the T3SS gene *tyeA* of *V. alginolyticus*, explored its biology and pathogenicity, and evaluated its effect as a live attenuated vaccine. Zhou et al. (2013) found that the mutant of T3SS chaperone escort *vscO* of *V. alginolyticus* showed an attenuated swarming ability and a 10-fold decrease in the virulence to fish. However, the Δ*vscO* mutant showed no difference in the biofilm formation and ECPase activity. The authors concluded that *vscO* is associated with the flagella of *V. alginolyticus*. The knockout of *vscO* gene results in the decline of group movement ability and thus reduced virulence (Zhou et al., 2013). Another mutant of *V. alginolyticus*, Δ*sodB* did not show any difference in growth when compared with wild type strains HY9901. However, the Δ*sodB* mutant resulted in the formation of biofilm, increased SOD activity and toxicity, increased ECPase activity and sensitivity to hydrogen peroxide, a decreased group movement ability and decreased adhesion to cytokine-induced killer (CIK) cells (Yanyan et al., 2019). Even though the mutant of T3SS effector Hop revealed an attenuated swarming phenotype and a 2,600-fold decrease in the virulence to grouper, the HY9901Δ*hop* mutant showed no difference in morphology, growth, biofilm formation and ECPase activity (Pang et al., 2018). Similarly in the present study, there was no significant difference between HY9901Δ*tyeA* and wild type strains HY9901 in growth, biofilm formation and ECPase activity but the HY9901Δ*tyeA* mutant showed an attenuated swarming phenotype and a nearly 40-fold decrease in virulence to zebrafish. A polar flagellum and numerous lateral flagella contribute to the swimming and swarming motilities respectively, by which some bacteria access an appropriate niche inside the host after infection, as observed in *V. parahaemolyticus* (Enos-Berlage et al., 2005). Therefore, swarming ability is closely related to the virulence of some bacteria. Biofilm formation is a multicellular behavior through which bacteria colonize the surface of host tissues and tank surfaces and are thereby highly resistant to antibiotics and host immune defense mechanisms (Parsek and Singh, 2003; Verstraeten et al., 2008). Extracellular products (ECP) produced by bacteria include proteases, hemolysins and siderophores. As a metabolite of bacteria, extracellular proteases used as virulence factors by *V. alginolyticus* play an important role in the process of infection. Flagella assist in swimming and can help bacteria enter the appropriate niche in the host after *Vibrio spp*. infection. Motility is an important index used to judge the virulence of bacteria (Watnick et al., 2001). Numerous studies have shown that flagellin is essential for flagellum formation, trivial movement and symbiosis, and it also greatly affects the colonizing ability of *Vibrio spp*. (Millikan and Ruby, 2004). The results of this study showed that there was no significant difference in bacterial growth rate, biofilm, and extracellular enzyme activity between the *tyeA* knockout and the wild strain of *V. alginolyticus*, suggesting that TyeA may not affect these characteristics in *V. alginolyticus*. Nevertheless, this study found that HY9901Δ*tyeA* had significantly decreased swarming motility, which may be associated with bacterial flagellar movement. Therefore, the *tyeA* gene may have a positive regulation in the swarming motility of *Vibrio alginolyticus*, and its regulatory mechanism needs further investigation.

It is generally agreed that T3SS can be secreted by bacteria *in vitro* or by contacting host cells (Buttner and Bonas, 2002; Blondel Carlos et al., 2016). The secretory pathway of T3SS *in vitro* and *in vivo* is complex, sometimes the regulation is completed by a single regulatory protein, and sometimes the regulation is completed by multiple interacting regulatory proteins. For example, the transcriptional expression of the T3SS1 gene cluster of *V. parahaemolyticus* in DMEM can be regulated by the regulatory proteins ExsA and ExsD. Among them, ExsA is a positive transcriptional regulation protein and ExsD is a negative transcriptional regulation protein. It was hypothesize that ExsA might play a direct role in the T3SS1 promoter sequence (Zhou et al., 2008) based on the discovery that ExsA protein directly binds the upstream region of effector protein VP1668 and VP1687 in gel retardation assay (EMSA). Furthermore, the co-expression of recombinant proteins labeled with different antigens showed that: the combination of ExsD and ExsA blocks the expression of T3SS1; the combination of ExsC and ExsD releases ExsA, thus allowing the expression of T3SS1 (Zhou et al., 2010). According to Miller et al. (2016), in a bile salt environment, VttRA and VttRB of *V.o cholerae* can regulate the secretion of T3SS structural genes and some effector proteins. In terms of *V. alginolyticus*, ExsA and ExsC play a positive regulatory role on the effector proteins Va1686 and Va1687, ExsD, and ExsE play a negative regulatory role, ExsA and ExsC act as stimulus to the swarming of the side flagella of *V. alginolyticus* and on the contrary, ExsD and ExsE act as hindrance to the swarming of the side flagella (Liu et al., 2016). Interestingly, the results of fluorescence quantification and drug susceptibility tests in this study indicated that TyeA negatively regulated T3SS protein (vscL, vscK and vscO are apparatus proteins, while vopN, vscN, and hop are effector or regulatory proteins) and drug resistance genes. However, the systematic research of the network regulatory mechanism of various *V. alginolyticus* regulatory proteins on effector proteins *in vitro* and *in vivo* is still needed. TyeA, a regulator in T3SS, is widely studied in other strains (Sundberg and Forsberg, 2003; Schubot et al., 2004; Amer et al., 2016), and some progress has been made (Joseph and Plano, 2007; Plano and Schesser, 2013). However, its regulation mechanism in *V. alginolyticu*s remains unknown. In order to further reveal the pathogenic mechanism of *V. alginolyticu*s, so as to achieve the purpose of controlling vibriosis, the research on the regulatory mechanism of the regulatory gene *tyeA* in *V. alginolyticus* will be followed-up in future investigations.

Moreover, although zebrafish will not specifically be the target of the vaccine in the future, studies have shown that zebrafish are a good model for teleost immune responses (Streisinger et al., 1981). For example, Zhang et al. (2012) used zebrafish to investigate the immune response following administration of a live attenuated *V. anguillarum* vaccine. Hua and co-author's results showed that Th17 cells were activated following vaccination of zebrafish (Hua et al., 2013). At the same time, a large number of studies have shown that zebrafish can be infected by marine vibrios (O'toole et al., 2004; Rojo et al., 2007; Rodríguez et al., 2008). Therefore, it is feasible to use zebrafish to study the effectiveness of a marine *Vibrio* vaccine. In this study, genes associated with innate immunity or inflammatory factors: IL-1 β, IL6, IL8, and TNF- α were studied. Additionally the expression profiles of bacterial flagellum recognition factor TLR5, adaptive immune factors including rag-1, and immunoglobulin IgM were assessed, as well as blood corpuscle specific marker genes c/elbp. In summary, the results show that HY9901Δ*tyeA* can effectively induce the protective immune response of zebrafish associated with pro-inflammatory and immunoglobulin activity. *V. alginolyticus* has been resistant to most antibiotics according to the results of drug sensitivity testing in this study. Antibiotic resistance is closely linked to antibiotic abuse (Chee-Sanford et al., 2001; Burridge et al., 2010). In the future, the direction for the prevention and control of aquatic diseases such as Vibriosis is to develop *Vibrio* vaccine and thereby reduce the use of antibiotics, which has great significance for protecting the environment and developing aquaculture sustainably. In the current study, the relative percentage survival rate of zebrafish immunized with the vaccine candidate strain Δ*tyeA* was 71.2%, which indicated that the construction of the deletion strain could be used as one of the effective ways to develop a vaccine against this pathogen. It provides an experimental basis for the prevention and control of *Vibrio spp*. in aquaculture. Studies have shown that compared with inactivated vaccines, live attenuated vaccines have the advantages of long-lasting efficacy and inducing strong cellular immunity, which is in line with the current needs of aquaculture, however their use is not permitted under current regulations in Europe.

## Conclusion

To sum up, we have successfully constructed the deletion strain of HY9901Δ*tyeA*. We found that the HY9901Δ*tyeA* strain exhibited decreased virulence to zebrafish, but the wild strain and the mutant strains were resistant to most antibiotics. The attenuated live vaccine was highly protective against *V. alginolyticus* and can induce protective immune responses in zebrafish. These results provide further evidence for the importance of T3SS in *V. alginolyticus* and provide reference for further study of this virulence factor. The immune effect and safety of the attenuated live vaccine will be tested and evaluated in aquaculture species in order to provide further theoretical basis and technical support for its application for the prevention and control of fish diseases caused by *V. alginolyticus*.

## Data Availability Statement

The datasets generated for this study can be found in the NCBI Accession No. MN328351.

## Ethics Statement

The animal study was reviewed and approved by Guangli Li and Guangdong Ocean University of ethics committee.

## Author Contributions

SZ and HP designed the experiments. SZ, XT, and JL generated experimental data and wrote the manuscript. HP, RH, SM, and JJ conceived the work and critically reviewed the manuscript. All authors contributed extensively to the work presented in this manuscript.

## Conflict of Interest

The authors declare that the research was conducted in the absence of any commercial or financial relationships that could be construed as a potential conflict of interest.

## References

[B1] Amer A. A. Gurung Jyoti M. Costa Tiago R. D. Kristina R. Zavialov A. V. Forsberg A.. (2016). TyeA hydrophobic contacts required for regulating ysc-yop type, III secretion activity by yersinia pseudotuberculosis. Front. Cell. Infect. Microbiol. 6:66. 10.3389/fcimb.2016.00066 27446813 PMC4914553

[B2] Anand S. Tomoko K. Galán J. E (2003). Synthesis and localization of the *Salmonella* SPI-1 type III secretion needle complex proteins PrgI and PrgJ. J. Bacteriol. 185, 3480–3883. 10.1128/JB.185.11.3480-3483.2003 12754250 PMC155383

[B3] Bauer A. W. Kirby W. M. Sherris J. C. Turck M. (1966). Antibiotic susceptibility testing by a standardized single disk method. Am. J. Clin. Pathol. 45, 493–496. 10.1093/ajcp/45.4_ts.493 5325707

[B4] Ben Kahla-Nakbi A. Chaieb K. Bakhrouf A. (2009). Investigation of several virulence properties among *Vibrio* alginolyticus strains isolated from diseased cultured fish in Tunisia. Dis. Aquat. Organ. 86, 21–28. 10.3354/dao02091 19899346

[B5] Blondel Carlos J. Park Joseph S. Hubbard Troy P. Pacheco Alline R. Kuehl Carole J. Walsh Michael J.. (2016). CRISPR/Cas9 screens reveal requirements for host cell sulfation and fucosylation in bacterial type III secretion system-mediated cytotoxicity. Cell Host Microbe 20, 226–237. 10.1016/j.chom.2016.06.010 27453484 PMC4982808

[B6] Burridge L. Weis J. S. Cabello F. Pizarro J. Bostick K. (2010). Chemical use in salmon aquaculture: a review of current practices and possible environmental effects. Aquaculture 306, 7–23. 10.1016/j.aquaculture.2010.05.020

[B7] Buttner D. Bonas U. (2002). Getting across–bacterial type III effector proteins on their way to the plant cell. EMBO J. 21, 5313–5322. 10.1093/emboj/cdf536 12374732 PMC129068

[B8] Cai S. H. Wu Z. H. Jian J. C. Lu Y. S. (2007). Cloning and expression of gene encoding the thermostable direct hemolysin from *Vibrio* alginolyticus strain HY9901, the causative agent of vibriosis of crimson snapper (*Lutjanus erythopterus*). J. Appl. Microbiol. 103, 289–296. 10.1111/j.1365-2672.2006.03250.x 17650188

[B9] Cai X. H. Peng Y. H. Wang Z. C. Huang T. Xiong X. Y. Huang Y. C.. (2016). Characterization and identification of streptococci from golden pompano in China. Dis. Aquat. Organ. 119, 207–217. 10.3354/dao02998 27225204

[B10] Chang C. C. Yeh M. S. Lin H. K. Cheng W. (2008). The effect of *Vibrio* alginolyticus infection on caspase-3 expression and activity in white shrimp *Litopenaeus* vannamei. Fish Shellfish Immunol. 25, 672–678. 10.1016/j.fsi.2008.09.004 18840531

[B11] Chee-Sanford J. C. Aminov R. I. Krapac I. J. Garrigues-Jeanjean N. Mackie R. I. (2001). Occurrence and diversity of tetracycline resistance genes in lagoons and groundwater underlying two swine production facilities. Appl. Environ. Microbiol. 67, 1494–1502. 10.1128/AEM.67.4.1494-1502.2001 11282596 PMC92760

[B12] Cheng L. W. Schneewind O. (2000). Yersinia enterocolitica TyeA, an intracellular regulator of the type III machinery, is required for specific targeting of YopE, YopH, YopM, and YopN into the cytosol of eukaryotic cells. J. Bacteriol. 182, 3183–3190. 10.1128/JB.182.11.3183-3190.2000 10809698 PMC94505

[B13] Dorothea R. A. Enrica B. Selma M. Da-Kang S. Xia L. Maria P. (2017). Steps for shigella gatekeeper protein mxic function in hierarchical type III secretion regulation. J. Biol. Chem. 292, 1705–1723. 10.1074/jbc.M116.746826 27974466 PMC5290946

[B14] Edwards R. A. Keller L. H. Schifferli D. M. (1998). Improved allelic exchange vectors and their use to analyze 987P fimbria gene expression. Gene 207, 149–57. 10.1016/S0378-1119(97)00619-7 9511756

[B15] Enos-Berlage J. L. Guvener Z. T. Keenan C. E. Mccarter L. L. (2005). Genetic determinants of biofilm development of opaque and translucent *Vibrio* parahaemolyticus. Mol. Microbiol. 55, 1160–1182. 10.1111/j.1365-2958.2004.04453.x 15686562

[B16] Ferracci F. Schubot F. D. Waugh D. S. Plano G. V. (2005). Selection and characterization of Yersinia pestis YopN mutants that constitutively block Yop secretion. Mol. Microbiol. 57, 970–987. 10.1111/j.1365-2958.2005.04738.x 16091038

[B17] Hua Z. Chao F. Haizhen W. Minjun Y. Qin L. Qiyao W. (2013). Transcriptome profiling reveals Th17-like immune responses induced in zebrafish bath-vaccinated with a live attenuated *Vibrio anguillarum*. PLoS ONE 8:e73871 10.1371/journal.pone.0073871 24023910 PMC3762715

[B18] Iriarte M. Sory M. P. Boland A. Boyd A. P. Mills S. D. Lambermont I.. (1998). TyeA, a protein involved in control of Yop release and in translocation of Yersinia Yop effectors. EMBO J. 17, 1907–18. 10.1093/emboj/17.7.1907 9524114 PMC1170537

[B19] Jones E. H. Feldman K. A. Palmer A. Butler E. Blythe D. Mitchell C. S. (2013). Vibrio infections and surveillance in Maryland, 2002-2008. Public Health Rep. 128, 537–45. 10.1177/003335491312800613 24179265 PMC3804097

[B20] Joseph S. S Plano G. V. (2007). Identification of TyeA residues required to interact with YopN and to regulate Yop secretion. Adv. Exp. Med. Biol. 603, 235–345. 10.1007/978-0-387-72124-8_21 17966420

[B21] Jun W. Yu-Hong S. Xue-Heng Z. Chang-Hong L. Ming-Yun L. Jiong C. (2015). Molecular characterization of an IL-1β gene from the large yellow croaker (*Larimichthys crocea*) and its effect on fish defense against *Vibrio alginolyticus* infection. Dongwuxue Yanjiu. 36, 133–141. 10.13918/j.issn.2095-8137.2015.03.003 26018856 PMC4790688

[B22] Katharine K. Watnick I. P. (2003). Environmental determinants of *Vibrio* cholerae biofilm development. Appl. Environ. Microbiol. 69, 5079–88. 10.1128/AEM.69.9.5079-5088.2003 12957889 PMC194957

[B23] Khavong P. Lorena N. (2016). Yersinia type III effectors perturb host innate immune responses. World J. Biol. Chem. 7, 1–13. 10.4331/wjbc.v7.i1.1 26981193 PMC4768113

[B24] Li W. X. Yao Z. J. Sun L. N. Hu W. J. Cao J. J. Lin W. X.. (2016). Proteomics analysis reveals a potential antibiotic cocktail therapy strategy for aeromonas hydrophila infection in biofilm. J. Proteome Res. 15, 1810–1820. 10.1021/acs.jproteome.5b01127 27110028

[B25] Li X. C. Xiang Z. Y. Xu X. M. Yan W. H. Ma J. M. (2009). Endophthalmitis caused by vibrio alginolyticus. J. Clin. Microbiol. 47, 3379–3381. 10.1128/JCM.00722-09 19710275 PMC2756916

[B26] Liu J. Lu S. Y. Orfe L. H. Ren C. H. Hu C. Q. Call D. R.. (2016). ExsE is a negative regulator for T3SS gene expression in vibrio alginolyticus. Front. Cell Infect. Microbiol. 6:177. 10.3389/fcimb.2016.00177 27999769 PMC5138213

[B27] Martinez-Argudo I. Blocker A. J. (2010). The *Shigella* T3SS needle transmits a signal for MxiC release, which controls secretion of effectors. Mol. Microbiol. 78, 1365–1378. 10.1111/j.1365-2958.2010.07413.x 21143311 PMC3020320

[B28] Masato S. Olga D. Mekalanos J. J. (2014). Vibrio cholerae T3SS effector VopE modulates mitochondrial dynamics and innate immune signaling by targeting Miro GTPases. Cell Host Microbe 16, 581–591. 10.1016/j.chom.2014.09.015 25450857 PMC4391628

[B29] Miller K. A. Sofia M. K. Weaver J. W. A. Seward C. H. Dziejman M. (2016). Regulation by ToxR-like proteins converges on vttRB expression to control type 3 secretion system-dependent Caco2-BBE cytotoxicity in vibrio cholerae. J. Bacteriol. 198, 1675–1682. 10.1128/JB.00130-16 27021561 PMC4959287

[B30] Millikan D. S. Ruby E. G. (2004). *Vibrio fischeri* flagellin a is essential for normal motility and for symbiotic competence during initial squid light organ colonization. J. Bacteriol. 186, 4315–4325. 10.1128/JB.186.13.4315-4325.2004 15205434 PMC421587

[B31] Nguyen L. Paulsen I. T. Tchieu J. Hueck C. J. Saier M. H. (2000). Phylogenetic analyses of the constituents of Type III protein secretion systems. J. Mol. Microbiol. Biotechnol. 2, 125–44. 10939240

[B32] O'toole R. Hofsten J. V. Rosqvist R. Olsson P.-E. Wolf-Watz H. (2004). Visualisation of zebrafish infection by GFP-labelled *Vibrio* anguillarum. Microbial. Pathog. 37, 41–6. 10.1016/j.micpath.2004.03.001 15194159

[B33] Pallen M. J. Beatson S. A. Bailey C. M. (2005). Bioinformatics analysis of the locus for enterocyte effacement provides novel insights into type-III secretion. BMC Microbiol. 5:9. 10.1186/1471-2180-5-9 15757514 PMC1084347

[B34] Pang H. Chen L. Hoare R. Huang Y. Zaohe W. Jian J. (2016). Identification of DLD, by immunoproteomic analysis and evaluation as a potential vaccine antigen against three *Vibrio* species in *Epinephelus* coioides. Vaccine 34, 1225–31. 10.1016/j.vaccine.2015.11.001 26562319

[B35] Pang H. Y. Qiu M. S. Zhao J. M. Hoare R. Monaghan S. J. Song D. W.. (2018). Construction of a *Vibrio* alginolyticus hopPmaJ (hop) mutant and evaluation of its potential as a live attenuated vaccine in orange-spotted grouper (*Epinephelus coioides*). Fish Shellfish Immunol. 76, 93–100. 10.1016/j.fsi.2018.02.0 29427720

[B36] Parsek M. R. Singh P. K. (2003). Bacterial biofilms: an emerging link to disease pathogenesis. Annu. Rev. Microbiol. 57, 677–701. 10.1146/annurev.micro.57.030502.090720 14527295

[B37] Plano G. V. Schesser K. (2013). The *Yersinia pestis* type III secretion system: expression, assembly and role in the evasion of host defenses. Immunol. Res. 57, 237–245. 10.1007/s12026-013-8454-3 24198067

[B38] Reed L. J. Muench H. (1938). A simple method of estimating fifty per cent endpoints12. Am. J. Epidemiol. 27, 493–497. 10.1093/oxfordjournals.aje.a118408

[B39] Rodríguez I. Novoa B. Figueras A. (2008). Immune response of zebrafish (*Danio rerio*) against a newly isolated bacterial pathogen *Aeromonas* hydrophila. Fish Shellfish Immunol. 25, 239–49. 10.1016/j.fsi.2008.05.002 18640853

[B40] Rojo I. De Ilarduya O. M. Estonba A. Pardo M. A. (2007). Innate immune gene expression in individual zebrafish after listonella anguillarum inoculation. Fish Shellfish Immunol. 23, 1285–1293. 10.1016/j.fsi.2007.07.002 17804254

[B41] Rubirés X. Saigi F. Piqué N Climent N. Merino S. Albertí S.. (1997). A gene (wbbL) from *Serratia marcescens* N28b (O4) complements the rfb-50 mutation of *Escherichia coli* K-12 derivatives. J. Bacteriol. 179, 7581–7586. 10.1128/JB.179.23.7581-7586.1997 9393727 PMC179713

[B42] Sadok K. Mejdi S. Nourhen S. Amina B. (2013). Phenotypic characterization and RAPD fingerprinting of *Vibrio* parahaemolyticus and *Vibrio* alginolyticus isolated during Tunisian fish farm outbreaks. Folia Microbiol. 58, 17–26. 10.1007/s12223-012-0174-x 22684973

[B43] Schubot F. D. Jackson M. W. Penrose K. J. Cherry S. Tropea J. E. Plano G. V.. (2004). Three-dimensional structure of a macromolecular assembly that regulates type III secretion in yersinia pestis. J. Mol. Biol. 346, 1147–1161. 10.1016/j.jmb.2004.12.036 15701523

[B44] Sganga G. Cozza V. Spanu T. Spada P. L. Fadda G. (2009). Global climate change and wound care: case study of an off-season *Vibrio alginolyticus* infection in a healthy man. Ostomy Wound Manage. 55, 60–62. 19387097

[B45] Simon R. Priefer U. Pühler A. (2019). A broad host range mobilization system for *in vivo* genetic engineering: transposon mutagenesis in gram negative bacteria. Nat. Biotech. 1, 784–791. 10.1038/nbt1183-784

[B46] Streisinger G. Walker C. Dower N. Knauber D. Singer F. (1981). Production of clones of homozygous diploid zebra fish (*Brachydanio rerio*). Nature 291, 293–296. 10.1038/291293a0 7248006

[B47] Sullivan C. Kim C. H. (2008). Zebrafish as a model for infectious disease and immune function. Fish Shellfish Immunol. 25, 341–350. 10.1016/j.fsi.2008.05.005 18640057

[B48] Sundberg L. Forsberg A. (2003). TyeA of *Yersinia pseudotuberculosis* is involved in regulation of Yop expression and is required for polarized translocation of Yop effectors. Cell. Microbiol. 5, 187–202. 10.1046/j.1462-5822.2003.00267.x 12614462

[B49] Thompson F. L. Iida T. Swings J. (2004). Biodiversity of vibrios. Microbiol. Mol. Biol. Rev. 68, 403–431. 10.1128/MMBR.68.3.403-431.2004 15353563 PMC515257

[B50] Verstraeten N. Braeken K. Debkumari B. Fauvart M. Fransaer J. Vermant J.. (2008). Living on a surface: swarming and biofilm formation. Trends Microbiol. 16, 496–506. 10.1016/j.tim.2008.07.004 18775660

[B51] Watnick P. I. Lauriano C. M. Klose K. E. Croal L. Kolter R. (2001). The absence of a flagellum leads to altered colony morphology, biofilm development and virulence in *Vibrio* cholerae O_139_. Mol. Microbiol. 39, 223–35. 10.1046/j.1365-2958.2001.02195.x 11136445 PMC2860545

[B52] Windle H. J. Kelleher D. (1997). Identification and characterization of a metalloprotease activity from *Helicobacter pylori*. Infect. Immun. 65, 3132–3137. 10.1128/IAI.65.8.3132-3137.1997 9234765 PMC175442

[B53] Xu Z. Wang Y. Han Y. Chen J. Zhang X.-H. (2010). Mutation of a novel virulence-related gene mltD in *Vibrio anguillarum* enhances lethality in zebra fish. Res. Microbiol. 162, 144–150. 10.1016/j.resmic.2010.08.003 21070855

[B54] Yanyan C. Fenglei W. Huanying P. Jufen T. Shuanghu C. Jichang J. (2019). Superoxide dismutase B (sodB), an important virulence factor of *Vibrio* alginolyticus, contributes to antioxidative stress and its potential application for live attenuated vaccine. Fish Shellfish Immunol. 89, 354–360. 10.1016/j.fsi.2019.03.061 30959182

[B55] Yonghong Y. Huanying P. Zejun Z. Dawei S. Yu D. Jichang J. (2016). Construction of live attenuated vaccine against *Vibrio alginolyticus* and the evaluation on its immunoprotection effect. Anim. Husbandry Feed Sci. 8, 85–93. 10.19578/j.cnki.ahfs.2016.02.007

[B56] Zhang Z. Wu H. Xiao J. Wang Q. Liu Q. Zhang Y. (2012). Immune responses of zebrafish (*Danio rerio*) induced by bath-vaccination with a live attenuated *Vibrio anguillarum* vaccine candidate. Fish Shellfish Immunol. 33, 36–41. 10.1016/j.fsi.2012.03.031 22507197

[B57] Zhou X. Konkel M. E. Call D. R. (2010). Regulation of type III secretion system 1 gene expression in *Vibrio parahaemolyticus* is dependent on interactions between ExsA, ExsC, and ExsD. Virulence 1, 260–272. 10.4161/viru.1.4.12318 21178451 PMC3073295

[B58] Zhou X. Shah D. H. Konkel M. E. Call D. R. (2008). Type III secretion system 1 genes in *Vibrio parahaemolyticus* are positively regulated by ExsA and negatively regulated by ExsD. Mol. Microbiol. 69, 747–764. 10.1111/j.1365-2958.2008.06326.x 18554322 PMC2610376

[B59] Zhou Z. Pang H. Ding Y. Cai J. Huang Y. Jian J.. (2013). VscO, a putative T3SS chaperone escort of *Vibrio alginolyticus*, contributes to virulence in fish and is a target for vaccine development. Fish Shellfish Immunol. 35, 1523–31. 10.1016/j.fsi.2013.08.017 23994282

[B60] Zujie Y. Zhuang G. Yuqian W. Wanxin L. Yuying F. Yuexu L. (2019). Integrated succinylome and metabolomics profiling reveals crucial role of S-ribosylhomocysteine lyase in quorum sensing and metabolism of *Aeromonas hydrophila*. Mol. Cell. Proteomics 18, 200–215. 10.1074/mcp.RA118.001035 30352804 PMC6356075

